# Unequal allocation of overt and covert attention in Multiple Object Tracking

**DOI:** 10.3758/s13414-022-02501-7

**Published:** 2022-05-13

**Authors:** Veronica Hadjipanayi, Andria Shimi, Casimir J. H. Ludwig, Christopher Kent

**Affiliations:** 1 School of Psychological Science, University of Bristol, Bristol, UK; 2 Department of Psychology, University of Cyprus, Nicosia, Cyprus

**Keywords:** Overt and covert attention, Multiple object tracking, Unequal attention allocation, Eye movements, Peripheral vision

## Abstract

In many real-life contexts, where objects are moving around, we are often required to allocate our attention unequally between targets or regions of different importance. However, typical multiple object tracking (MOT) tasks, primarily investigate *equal* attention allocation as the likelihood of each target being probed is the same. In two experiments, we investigated whether participants can allocate attention *unequally* across regions of the visual field, using a MOT task where two regions were probed with either a high and low or with equal priority. Experiment 1 showed that for high-priority regions, accuracy (for direction of heading judgments) improved, and participants had more frequent and longer fixations in that region compared with a low-priority region. Experiment 2 showed that eye movements were functional in that they slightly improved accuracy when participants could freely move their eyes compared with when they had to centrally fixate. Replicating Experiment 1, we found better tracking performance for high compared with low-priority regions, in both the free and fixed viewing conditions, but the benefit was greater for the free viewing condition. Although unequal attention allocation is possible without eye movements, eye movements seem to improve tracking ability, presumably by allowing participants to fixate more in the high-priority region and get a better, foveal view of the objects. These findings can help us better understand how observers in real-life settings (e.g., CCTV monitoring, driving) can use their limited attentional capacity to allocate their attention unequally in a demand-based manner across different tracking regions.

Living in a dynamic environment, the ability to allocate attention to multiple objects simultaneously, and even unequally, is a cognitive skill that is often required during different everyday tasks (e.g., sports, driving, video gaming) and safety critical tasks (e.g., CCTV monitoring, lifeguards monitoring a pool, air traffic control). It has been found that drivers and athletes who exhibit more efficient allocation of eye movements have better driving and sports performance, respectively (Jacobson & Matthaeus, 2014; Mackenzie & Harris, 2017; Memmert, 2009). The efficacy of attention allocation can be improved if practiced often (Allen et al., 2004; Green & Bavelier, 2003; Romeas et al., 2016), with some evidence suggesting that these practice effects can even generalize beyond the trained task, improving decision-making processes (Romeas et al., 2016) and allocation of spatial attention in untrained locations and tasks (Romeas et al., 2016). Therefore, it is important to investigate how attention is allocated between different targets or regions, and the role of eye movements in this process, in order to better understand the cognitive mechanisms of attention allocation and find ways to improve the efficiency with which we perform many real-life tasks.

The multiple object tracking (MOT; Pylyshyn & Storm, 1988) task has been used extensively to investigate the processes of dynamic attention allocation in a laboratory environment (Cavanagh & Alvarez, 2005; Meyerhoff et al., 2017). This task addresses the central question of how attention is allocated in a dynamic visual scene with multiple moving stimuli (Huang et al., 2012; Kunar et al., 2010). In a typical MOT task, participants are asked to track some objects, initially indicated as targets, amongst visually similar distractors for a short period of time. At the end of a trial, movement of objects ceases, and while all objects are visible on-screen participants, are asked to report the status of one object (i.e., whether it was a target or distractor). Alternatively, upon movement ceasing, participants might be asked to report the trajectory of a queried target or location of a target in a highlighted region, immediately before the screen went blank (Howard et al., 2017), providing a continuous measure of tracking performance. Tracking performance on MOT tasks can be affected by a range of different factors which influence the tracking load like, the speed of movement (Alvarez & Franconeri, 2007), the hemifield of presentation (Alvarez & Cavanagh, 2005), the proximity of objects (Franconeri et al., 2008; Tombu & Seiffert, 2008) or the number of moving objects (Drew et al., 2011; Yantis, 1992). Participants seem able to track up to four objects simultaneously, with tracking performance decreasing as the number of to-be-tracked objects increases beyond this (Intriligator & Cavanagh, 2001; Scholl et al., 2001), although at slower speeds it seems up to eight objects can be tracked simultaneously (Alvarez & Franconeri, 2007). These limitations to tracking performance suggest that our attentional resource is finite as tracking accuracy decreases with an increasing tracking load.

The role of foveal and peripheral vision during attentional tracking has been studied in different environments including MOT tasks. Landry et al. (2001) investigated the eye movements of participants when they monitored objects for potential collisions during a simulated air-traffic control tracking task. Results indicated increased saccades when participants monitored targets on a potential collision course compared with when they monitored other targets that were not likely to collide. This evidence indicates that observers tend to fixate on items, particularly when tracking gets difficult. This suggests that making eye movements to targets facilitates tracking performance as saccades can allow for a foveal view of objects, which can in turn aid in updating their exact location. In this context, Zelinsky and Todor (2010) investigated the role of ‘rescue saccades’ in MOT, which refer to saccades initiated when tracking load increases (e.g., when the target is close to a distractor), highlighting the importance of overt attention and the oculomotor system in events that might cause temporary loss of tracking (e.g., during occlusion).

However, the importance of *covert* attention and peripheral vision during attentional tracking has also been established, suggesting that what we fixate is not necessarily what we attend to. In particular, it has been found that task-relevant stimuli can be detected and processed when they appear both inside and outside the fixation region (Lichtenstein-Vidne et al., 2007; Linnell & Humphreys, 2004). However, evidence suggests that observers tend to rely on peripheral vision at lower tracking loads and switch to foveal vision when tracking demands increase (Zelinsky & Neider, 2008).

Vater et al. (2016) investigated whether peripheral vision can be used to track multiple moving objects and detect single-target changes. Their results indicated that peripheral vision is naturally used to detect changes in motion and form. Taking it further, Vater et al. (2017b) reported that detection of changes in form and motion is faster when changes occur close to the fixation region. If the location of fixation is further away from the location of target change, motion changes are still detected with the same accuracy while form changes are less accurately detected. This suggests that peripheral vision is more sensitive to changes in motion than in form. The use of peripheral vision for target motion and form detection has also been replicated in sports settings using simulated environments (Vater, 2019; Vater et al., 2017a). Taken together, these studies provide evidence for the plausibility of using peripheral vision to track multiple moving targets and to detect motion and form changes in MOT tasks.

The majority of MOT tasks can be seen as traditional equal attention allocation tasks. However, this is unlike many real-world settings where observers must often allocate their attention unequally across different individual targets or regions of the visual field that are associated with different levels of importance. For example, a driver is required to allocate attention unequally between targets of higher importance (e.g., other vehicles and pedestrians) and targets of lesser, yet not completely negligible, importance (e.g., road signs). To our knowledge, only a few studies have investigated unequal attention allocation, where different targets or regions are associated with different levels of priority.

Liu et al. (2005) modified a traditional MOT task by manipulating the speed of the objects such that half the objects moved at a fast (i.e., 6°/s) and half at a slow (i.e., 1°/s) speed. Although it was not part of the primary research aim, evidence in favour of unequal attention allocation was obtained as similar tracking performance was observed across slow- and fast-moving objects. Given that tracking becomes more difficult at higher speeds, this result suggests that participants allocated more attention to the objects that moved faster. Similarly, Chen et al. (2013) manipulated speed in a task where four pairs of discs were presented to the participant and each pair moved on a circular trajectory in each of the four quadrants of the screen. Results indicated that the speed limit for detecting a target is higher if a secondary target moves at a slow rather than at a fast speed. This finding suggests that when one target is moving at a slower speed, more attentional resource is left to be allocated to the faster moving target, providing evidence in favour of unequal attention allocation. A similar attentional bias towards tracking targets that are in close proximity to distractors was also observed by Meyerhoff et al. (2018) who investigated the influence of interobject spacing during MOT. These findings indicate that unequal attention allocation occurs in a stimulus-driven manner and can be advantageous to avoid confusion between targets and distractors in close proximity (Meyerhoff et al., 2016). Iordanescu et al. (2009) provided further evidence for unequal attention allocation by investigating participants’ ability to reallocate their attention during tracking. During the trial, the distance from each target to its closest distractor was calculated as the degree of crowding around each target. Observers allocated their attention unequally while tracking such that more attentional resource was devoted to crowded targets (i.e., targets with the shortest distance from distractors) that were at more risk of being confused with distractors, than to uncrowded targets.

In the studies reviewed above, evidence for unequal attention allocation was provided as a result of manipulations of different aspects of the task such as the objects’ speed (Chen et al., 2013; Liu et al., 2005) and the proximity between objects (Iordanescu et al., 2009; Meyerhoff et al., 2016). Evidence for unequal attention allocation has also been obtained in studies that involved direct manipulation of the *priority* of targets or certain features of the targets (e.g., location, identity, colour), which consequently requires participants to prioritize those target or features above other targets or features (Fitousi, 2016; Miller & Bonnel, 1994; Posner, 1980). These tasks provide evidence for the ability of participants to allocate their attention unequally in a *goal-directed* and strategic manner, based on the instructions of the task or on the priority assigned directly to target. For example, Cohen et al. (2011) used a modified multiple identity tracking (MIT) task, which typically involves tracking objects that have a unique identity (Oksama & Hyönä, 2008). When participants were asked to prioritize the location over the identity of the targets, they exhibited better position- versus identity-tracking performance, indicating unequal attention allocation to different features of the same object (Cohen et al., 2011). Crowe et al. (2019) directly manipulated priority in a modified MOT task to investigate whether participants could allocate their attention unequally between targets. Priority of targets was manipulated such that two objects were associated with two different probabilities of being probed, as signalled at the start of a trial, with the probabilities (as percentages) appearing on the object (e.g., 25 and 75; 50 and 50). These numbers, representing the likelihood of each of the two targets being queried about their status (i.e., position or trajectory), allowed the participants to prioritize the objects unequally (in the case of 25 and 75) or equally (as in standard MOT, in the case of 50 and 50). Results indicated improved tracking accuracy (i.e., lower magnitude of error) and lower guessing rates as the priority of the target increased. These findings provide evidence for goal-directed unequal attention allocation as top-down instructions led participants to allocate more attention to the high- versus low-priority targets.

However, Crowe et al. (2019) only inferred attention allocation from perceptual performance as no direct measures of attention were used (such as eye tracking; Meyerhoff et al., 2017). Additionally, since probed priorities were presented on the actual targets, the particular MOT task used by Crowe et al. (2019) has a component of MIT as well, as participants were required to assign a certain priority (which could be used as an identifier—e.g., ‘the high one’) to each target. This could have created identity–location bindings, which refer to perceptual associations created between a targets’ unique identity and its location (Howe & Ferguson, 2015; Oksama & Hyönä, 2008; Saiki, 2002). Identity encoding is a process that requires additional attentional resource and could have influenced attention allocation of participants (Cohen et al., 2011). It is important to explore goal-directed unequal attention allocation in a purer MOT task, in which individual targets are not assigned a unique identity to investigate how attention is allocated between distinct identical objects. This may be addressed in a modified MOT task, where different tracking regions, and not individual targets, are associated with a certain likelihood of being probed. In addition, measuring eye movements of participants may also be expected to elucidate how observers allocate their attention unequally across different regions.

The experiments reported in this article aimed to investigate whether participants can allocate their attention unequally across two regions of the visual field, in a modified trajectory-tracking MOT task where two distinct tracking regions were probed with high and low priority or equal priority. Trajectory-tracking MOT tasks have been characterized as a suitable measure of tracking performance as they require participants to respond by providing the direction of the queried target instead of providing a target vs distractor response like in traditional MOT tasks (Horowitz & Cohen, 2010; Howard et al., 2017).

In Experiment 1, we investigated whether attention can be allocated unequally across two regions of the visual field, by examining differences in accuracy with which participants report the direction of heading of an item probed in a low, equal, or high-priority region. In Experiment 2 we further investigated the functional role of eye movements in unequal attention allocation. Although the usefulness of peripheral vision for detecting target changes during MOT tasks has already been established (Vater et al., 2016, 2017a, b), the role of covert attention has not been investigated when attention is unequally allocated. We compared performance in free-viewing and fixed-viewing conditions to investigate (a) whether attention can be unequally allocated by relying solely on peripheral vision (i.e., fixed-viewing condition) and (b) which, if any, of the two viewing conditions, free (i.e., foveal tracking of objects) or fixed (i.e., peripheral tracking of objects), facilitates trajectory tracking in the current modified MOT task.

## Experiment 1

The aim of Experiment 1 was to measure directly, via eye tracking, attention allocation and investigate goal-directed unequal attention allocation in a MOT task that removes the possibility of identity–location bindings being formed. Ethical approval was obtained from the National Bioethics Committee of Cyprus (EEBK/EΠ/2020/26). The study was conducted according to the revised Declaration of Helsinki (2013). The aims and hypotheses of Experiment 1 were preregistered on the Open Science Framework and can be found online (https://osf.io/wkcj5/).

## Method

### Participants

Thirty-three individuals were recruited from the University of Cyprus and surrounding areas via the Experimental Credit Scheme and word of mouth. Testing of participants was carried out at the Centre of Applied Neuroscience (CAN), University of Cyprus. G*Power (Version 3.1; Faul et al., 2007) was used to calculate the required sample size for this experiment. Existing data from a pilot experiment indicated an effect size of *d≈* 1.14, for the difference in error of tracking means between the low- and high-priority conditions. Crowe et al. (2019) tested between 27 and 44 participants in their study. To be consistent with their work, we set a samples size of 33. This sample size gave us at least 95% power of detecting a similar effect size at an alpha of .05.^1^ Participants were required to have normal or corrected-to-normal vision and be less than 35 years old.

### Materials

The MOT task was programmed, and run, using MATLAB (2019a, The MathWorks, Natick, MA) and Psychtoolbox (Psychtoolbox-3.0.13; www.psychtoolbox.org). Stimuli were presented on a PC running Windows 7. A 24-in. BenQ monitor was used, with a resolution of 1,920 × 1,080 pixels, running at 60 Hz. The stimulus window was 1,200 × 900 pixels. At a viewing distance of 70 cm, 1° corresponds to 45 pixels. An EyeLink 1000+ (SR Research Ltd.) video-based tracker was used. The eyes were tracked at a sampling rate of 1000 Hz. The eye tracker was calibrated at the start of every block of trials (using the in-built 9-point calibration routine). Saccades and fixations were parsed offline using the velocity and acceleration criteria of 30°s^-1^ and 8000°s^-2^, respectively.

On every trial, eight black (RGB value: 0, 0, 0) discs with radius 1.14° of visual angle were presented on a mid-grey screen (RGB value: 128,128,128), four in the upper region and four in the lower region of the screen. The discs then moved randomly around the screen, with an elastic collision formula applied if two discs collided with each other and a reversal of velocity if a disc hit a boundary. All discs bounced on the midline separating the two screen regions so that no disc from one screen region could exit or enter the other region. Discs initially appeared on the screen at quasi-random locations, at least 2.53° from the boundaries and 1.52° from other discs and moved at an average speed of 10° per second. The duration of movement was randomly drawn from a uniform distribution with a range of 6-8 s. The centre of all disc positions averaged across all frames was approximately the centre of the screen.

### Design

The priority of screen regions (upper half and lower half) was manipulated in a within-subjects design with three levels: high (70%), equal (50%), and low (30%). On a given trial, the combined values total 100 so numbers were represented in three different combinations: 70-30 (i.e., 70 in the upper and 30 in the lower region of the screen), 50-50 or 30-70 (i.e., 30 in the upper and 70 in the lower region of the screen). These numbers represent the likelihood of the ‘queried’ item appearing in the upper or in the lower region of the screen, respectively. Three dependent variables were measured: tracking error, gaze time spent on each screen region, and gaze deviation from the centre. Tracking error was indexed by the relative difference (in degrees) between participants’ estimated direction of heading and the actual direction of heading of the item. Higher absolute values represent greater discrepancy between estimated and actual item heading and therefore represent greater error (where zero is perfect accuracy) so less tracking accuracy. Proportion of gaze time spent looking at each screen region was computed on the basis of all the gaze samples, excluding blinks. Note that as a result, gaze time includes fixations, saccades, and epochs of smooth pursuit. Gaze deviation from the centre was indexed as the vertical distance above or below the centre of the screen.

### Procedure

**Fig. 1:**
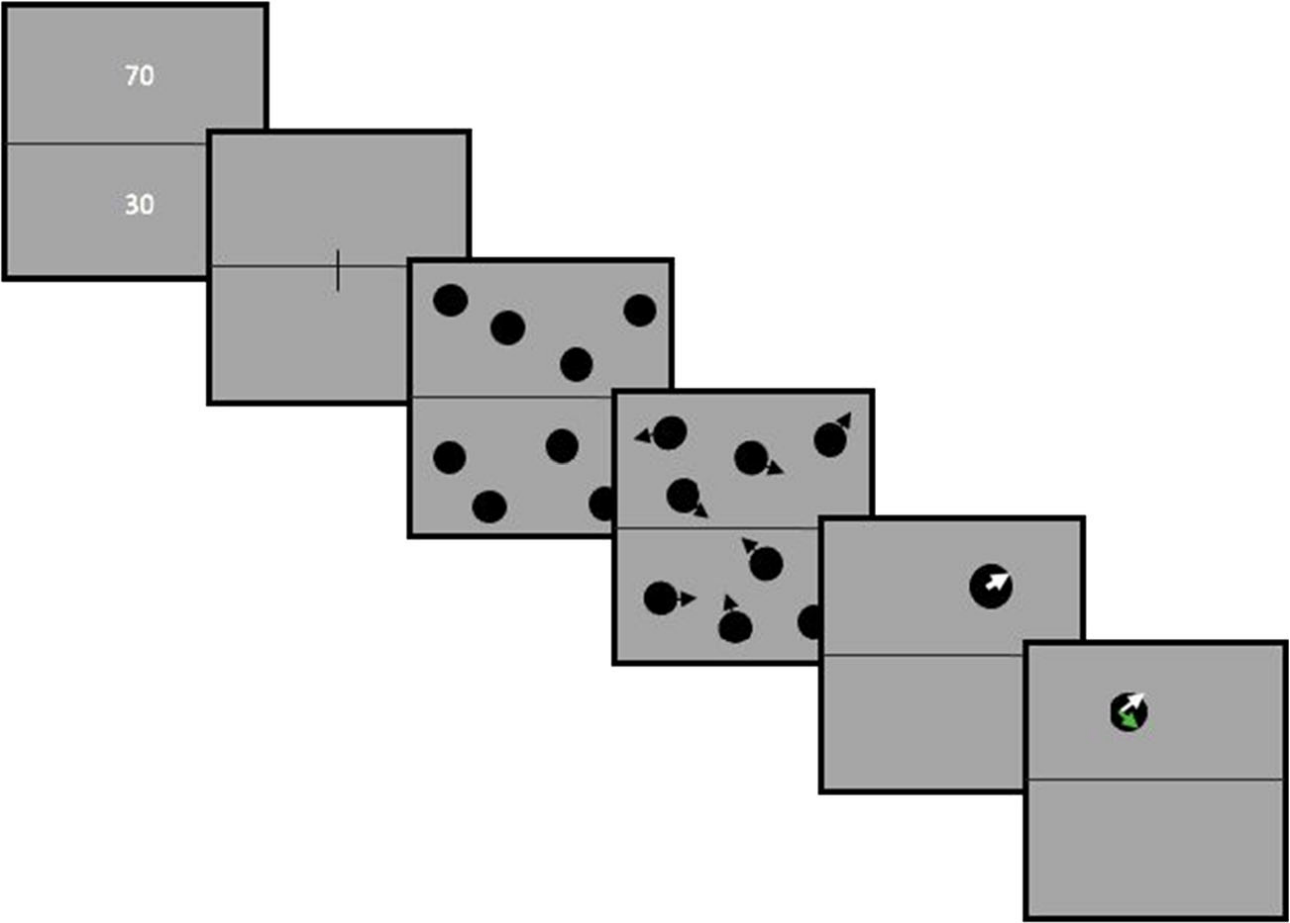
Trajectory tracking task timeline. At the beginning of the trial, numbers representing the likelihood of the “queried” item appeared in the upper or in the lower region of the screen, respectively. These numbers were either 70-30, 30-70 or 50-50. Then numbers disappeared and fixation line was presented. Subsequently, eight discs appeared on the screen, four in the upper and four in the lower regions of the screen. Then discs started moving around the screen without crossing the horizontal boundary (the black arrows were not presented on the screen but are used here to represent movement). After period of movement all discs disappeared, except one (which was either in the upper or in the lower screen region based on the probe probability of each region). Participants were then asked to click on the direction they thought the disc was going. After participants’ response, feedback was presented on the screen. A green arrow appeared indicating the target’s correct trajectory. (Colour figure online)

Figure 1 illustrates the timeline of a trial. A fixation screen appeared at the beginning of each trial and the experimenter initiated the trial upon accurate fixation. The fixation point was a vertical line of 0.4° of visual angle at the centre of the middle line dividing upper and lower screen regions. The intertrial interval was minimum 1,000 ms but often longer as it was dependent on the participant fixating accurately and the experimenter initiating the trial manually. Recording terminated at the end of every block of 30 trials.

Throughout the experiment, the screen was divided horizontally in two regions of equal area. At the beginning of each trial, before the discs appeared, the two likelihoods were presented on the screen for 3,000 ms, one in the upper and one in the lower region of the screen. For instance, in trials with the combination of 70 in the upper and 30 in the lower region of the screen, the ‘queried’ item that participants had to respond to, came from the upper region with a probability of 0.7 and from the lower region with a probability of 0.3. Participants were given clear instructions on what these numbers meant before starting the practice trial and had the opportunity to ask any questions.

Participants were instructed to keep tracking the discs while they were moving. At the end of each trial, all discs disappeared except one. The queried item would either be in the upper or in the lower region of the screen depending on the probed priority level assigned to each region. The participants’ task was to click, using the left mouse button, on the direction they thought this disc was moving. Participants first clicked inside the disc to ‘activate’ a “dial” on the disc with an arm of 1.14° extending from the item’s centre. The initial direction of the arm was set randomly. Participants then moved the arm (using the mouse) to indicate the estimated direction of travel and confirmed their answer with a second left mouse click. Feedback, consisting of a green arrow, of size 1.14° of visual angle, was given on each trial, indicating the correct direction of travel.

Participants completed 10 practice trials, followed by 150 experimental trials equally divided into 5 blocks of 30 trials. Within a block of 30 trials, there were 10 trials of each of the following types: 70-30; 30-70; 50-50 (upper – lower screen region). The frequencies of being probed in the upper or lower region of the screen followed the nominal probabilities—that is, 7-3; 3-7 and 5-5. Therefore, within a block there were 14 trials in which a target from the high-priority region was probed, 10 trials in which a target from the equal priority region was probed and six trials in which a target from the low-priority region was probed^2^. The order of trials was randomized for each participant. Hemifield presentation was counterbalanced across trials for every participant such that, the upper and lower screen regions were probed an equal number of times. The eye tracker was recalibrated before each block. The total testing time was approximately 60 minutes.

## Results

Linear mixed-effects models (LMEs; Baayen et al., 2008; Barr et al., 2013) were used to analyze the data using the lme4 package (Bates et al., 2015) for the R computing environment (R Core Team, 2015). Linear mixed-effects analysis was conducted with priority of screen regions entered as a fixed effect and a random intercept for subjects. Data for both perceptual performance and gaze measures were analyzed aggregated across trials to ensure that the observations were normally distributed. We report *p* values derived from a likelihood ratio test comparing the full model, including the predictor variable of priority, to the null model which included a random intercept for subjects only, without priority included.

### Planned analyses

#### Perceptual performance

**Fig. 2:**
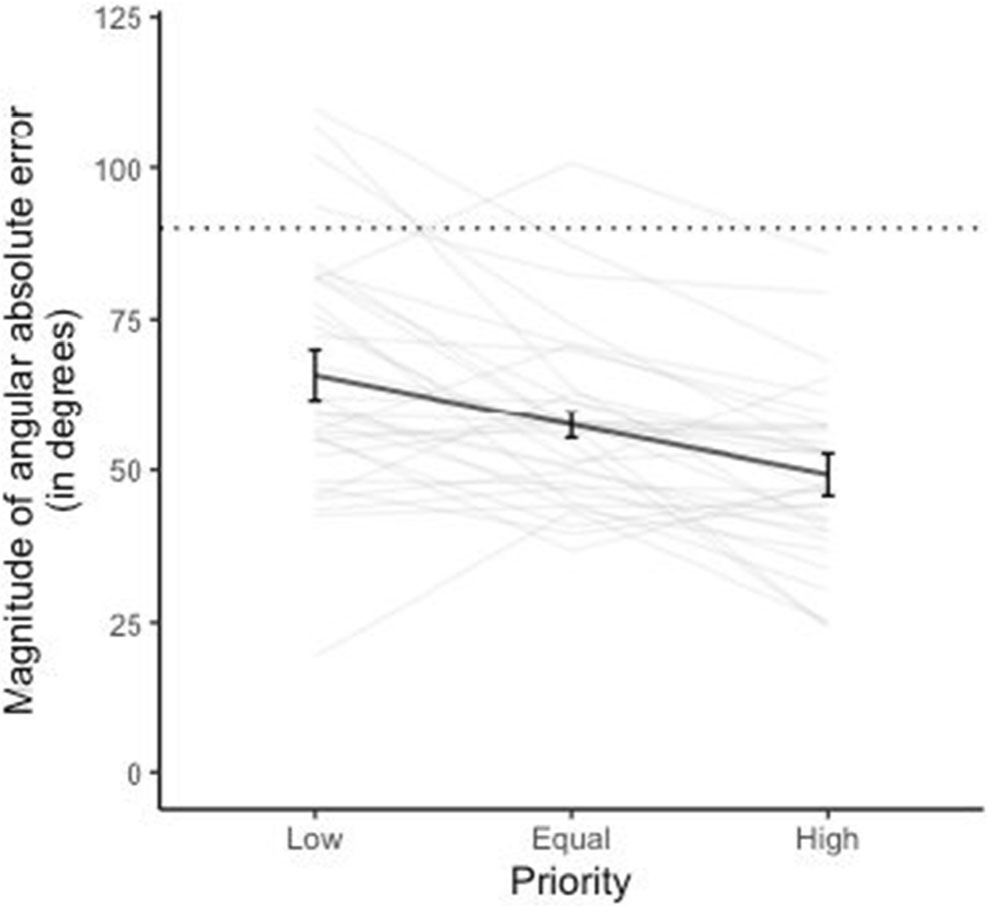
Magnitude of angular absolute error for each priority level. Black bold line indicates average magnitude of angular error across all 33 participants. Error bars represent 95% confidence intervals following Morey (2008). Grey lines indicate magnitude of angular error for each participant individually. Dashed line indicates the level of chance performance

Figure 2 indicates tracking performance of participants in all three priority conditions. If people responded completely randomly, we would expect an average absolute tracking error of 90°. Clearly, the majority of participants performed better than that. Moreover, tracking accuracy improved as priority increased. Specifically, there was a main effect of screen priority on magnitude of angular error, χ^2^(1) = 29.65, *p* < .001, whereby as the priority of screen region increased, the magnitude of angular error decreased, (*b* = −0.421, *SE* = 0.06, *t* = 6.12, *p *< .001).

Following the method of Crowe et al. (2019) and Horowitz and Cohen (2010; similar to Zhang & Luck, 2008), we conducted a model-based analysis to estimate the guessing rate and precision of tracking. A von Mises distribution (the circular equivalent of a normal distribution) centred on 0 was used to represent participants’ errors when the probed item was tracked successfully. A circular uniform distribution was used to represent participants’ responses when they lost track of the item and consequently guessed its direction. The MASS package (Venables & Ripley, 2002) was used for the fitdistr function and the circular package (Agostinelli & Lund, 2017) was used for the von Mises and circular uniform distributions functions.

**Fig. 3:**
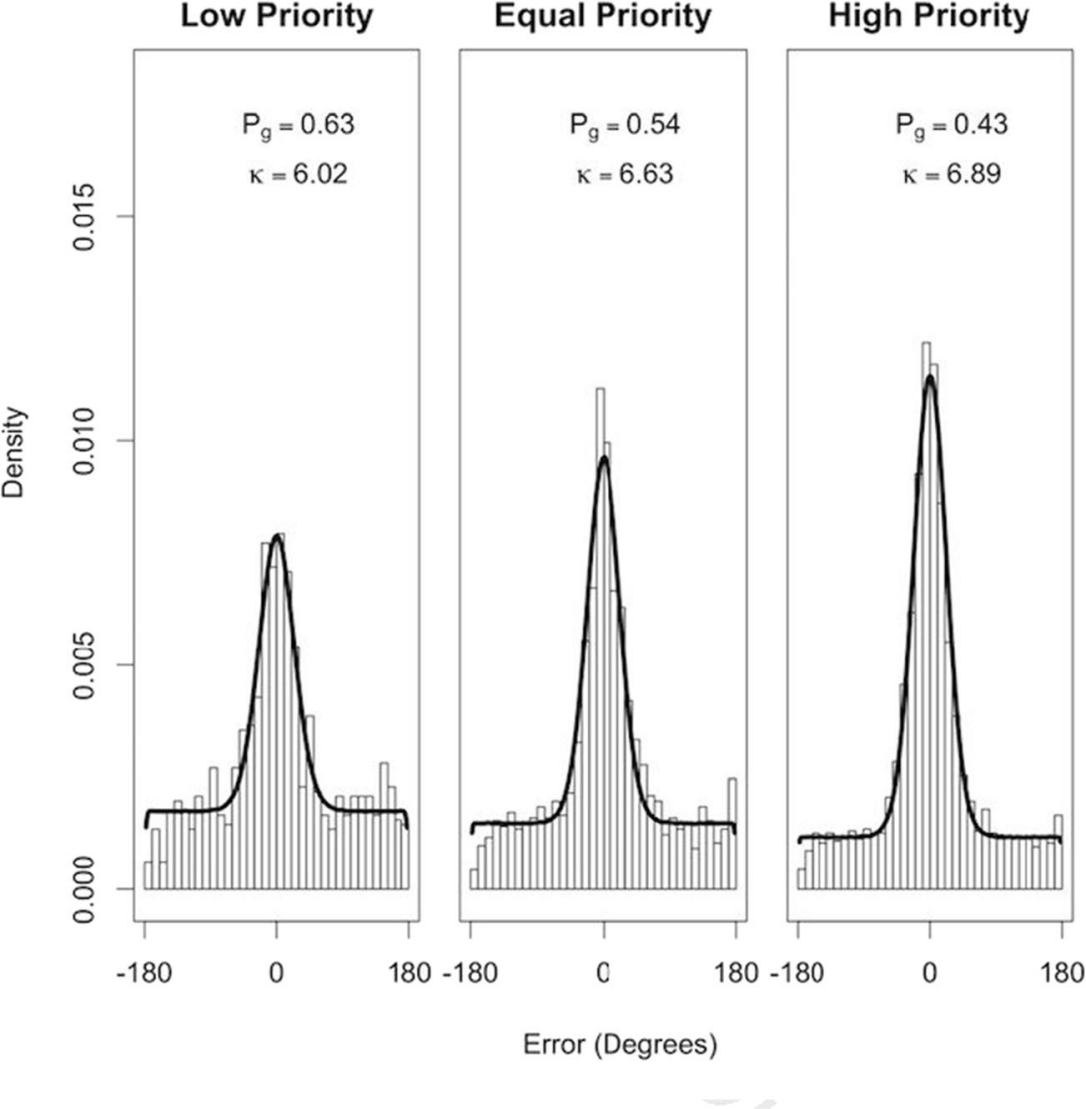
Mixture model fits for the combined data across participants at each of the three priority levels. The density plot demonstrates the actual data and the black density line illustrates the model fit. The best-fit parameters of the proportion of guessing (*P*_*G*_) and precision of tracking (*κ*) are also shown

Figure 3 shows the mixture model fits for error data pooled over all participants, at each of the three priority levels. The parameter *P*_*G*_ represents the probability of a random guess and the parameter *κ* represents tracking precision (the concentration of the von Mises component). The higher the *κ* value, the narrower the distribution around the mean, illustrating higher precision. The model fit is consistent with the analysis of error data above, illustrating that with increasing priority, the proportion of guessing decreases and precision increases, replicating the results of Crowe et al. (2019).^3^

#### Gaze measures

Two measures were drawn from the eye-tracking data: proportion of time spent by each participant looking at the upper screen region and the mean vertical distance (in degrees) from the centre of the screen, in each of the three different priority conditions (low, equal or high). These two measures were used to assess how participants allocate their overt attention during tracking across the two regions of the screen. It is worth noting that mean vertical distance from the centre is not a measure of how much participants *moved* their eyes during tracking, but rather a supplementary gaze measure for how overt attention was allocated across the two screen regions. The proportion of time and mean vertical distance, averaged across trials, were entered into the LME analysis in the same way as the magnitude of angular error.

**Fig. 4:**
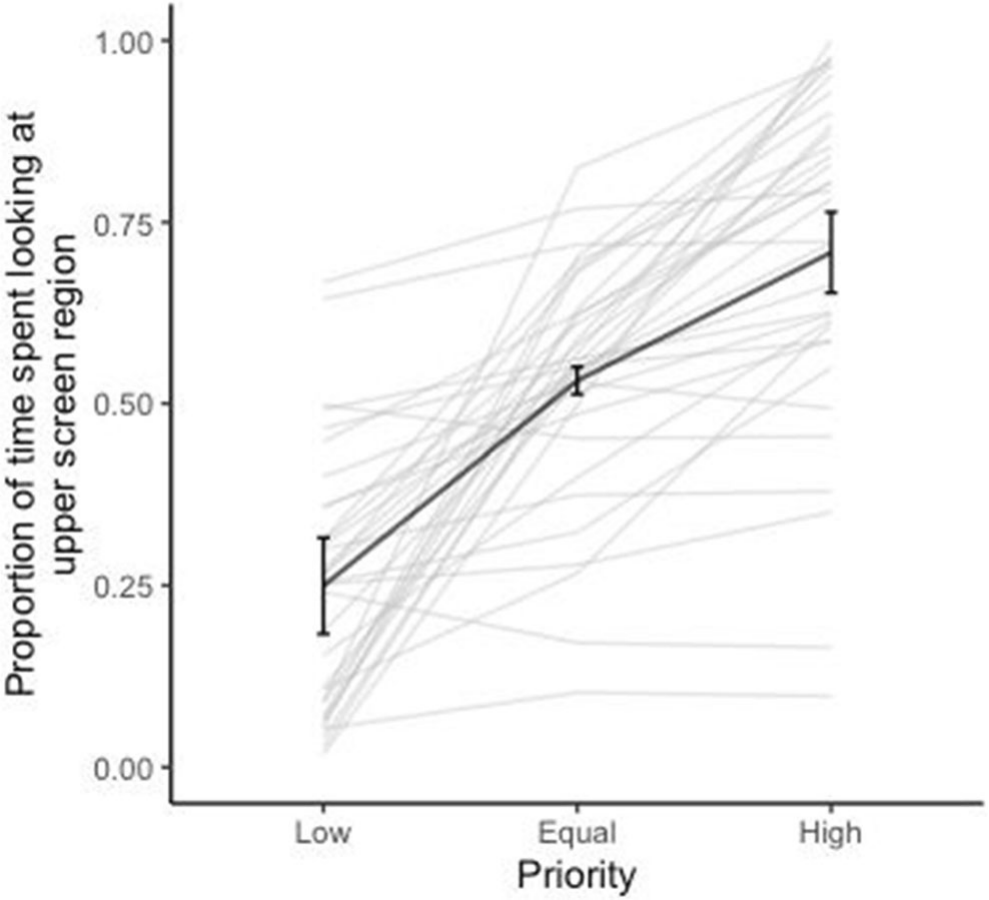
Proportion of time spent at the upper screen region, for each priority level presented. Black bold line indicates average proportion of time spent looking at the upper screen region across all 33 participants. Error bars represent 95% confidence intervals following Morey (2008). Grey lines indicate proportion of time spent looking at the upper screen region for each participant

Figure 4 indicates that the higher the priority a screen region was probed with, the more time was spent looking at that region. There was a significant effect of priority on proportion of time spent looking at upper screen region, χ^2^(1) = 69.09, *p* < .001. Participants spent more time looking at the upper region when it was more likely to be probed, (*b* = 0.011, *SE* = 0.001, *t* = 10.77, *p* < .001). Since Fig. 4 illustrates proportion of time spent looking at the upper screen region, it offers a reflection of the proportion of time spent looking at the lower region as well (i.e., proportion of time spent looking at lower region when probed with high priority is equal to 1 minus the proportion of time spent looking at upper region when probed with lower priority).

The finding that participants spent more time looking at a screen region that was probed with higher priority (Fig. 4), is further supported by the analysis of the mean vertical distance of eye gaze from the centre. Participants fixated, on average, higher up the screen when the upper region was probed with a higher priority and further down the screen when the upper region was probed with a lower priority. There was a significant effect of priority of the upper screen region on mean vertical distance from the centre, χ^2^(1) = 77.32, *p* < .001, with distance increasing as the upper screen region was more likely to be probed (*b* = 0.099, *SE* = 0.009, *t* = 10.82, *p* < .001). These findings provide further evidence for participants’ gaze behaviour being influenced by priority, suggesting that they were looking more at high versus low-priority regions.

### Exploratory analysis

**Fig. 5:**
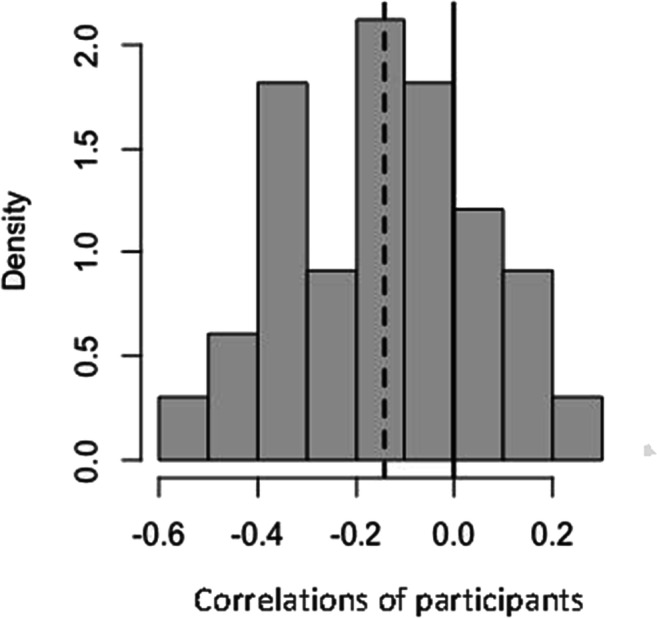
Histogram of correlations of individual participants between the proportion of time spent looking at the probed region and the tracking error (in degrees). The vertical bold line indicates 0 correlation and the vertical dotted line indicates the mean of all individual correlations.

An outstanding question is whether and to what extent the gaze bias influenced perceptual performance. Therefore, we assessed the relationship between the proportion of gaze time spent in the probed region with absolute tracking error. We computed the correlation for each individual participant at a trial level, pooled over the three conditions. Figure 5 shows the distribution of these correlations and demonstrates that 76% of the individual correlations are negative. The mean correlation across participants of −.14 was significantly different from 0, *t*(32) = −4.22, *p *< .001*. *This result indicates that the more the participants were looking on the probed screen region, the lower their absolute tracking error.

## Discussion

The perceptual performance and gaze data of Experiment 1 suggest that attention was allocated unequally between the two visual fields in a top-down fashion, with attention preferentially directed to high versus low-priority regions of the screen, as evidenced by improved tracking performance (Fig. 2), prolonged eye gaze (Figure 4), and greater distance from the horizontal midline. Participants seemed to allocate their attention and focus on high-priority regions of the screen during movement of objects, resulting in decreased angular error, decreased guessing rate, and increased precision of estimating the discs’ trajectory. This finding is a form of probability matching (Eriksen & Yeh, 1985) and supports the idea that participants devoted the majority of their attention to the high-priority region but did not completely neglect the low-priority regions. Current results replicate and extend those of Crowe et al. (2019), providing support that in a MOT task in which the objects are not individuated, participants are able to allocate their attention unequally between different tracking regions depending on the priority assigned to each region.

We used eye movements as a direct measure of attention allocation. However, there may not be a one-to-one mapping between the loci of attention and gaze. A dissociation for both reflexive (Hunt & Kingstone, 2003a) and voluntary (Hunt & Kingstone, 2003b) shifts between overt and covert attention is well established, supporting the possibility of shifting attention without shifting eye gaze (Kerr, 1971; Posner, 1980). However, *just prior to generating a saccade*, attention is focused on the future saccade target (Hoffman & Subramaniam, 1995; Juan et al., 2004; Kowler et al., 1995; Murthy et al., 2001; Sato & Schall, 2003; Schall, 2004). Only in that brief timeframe there appears to be an obligatory coupling between overt and covert attention. Participants in a MOT task can still attend to a target or specific region of the visual field using their peripheral vision, without moving their eyes (Vater et al., 2016, 2017a, b). Therefore, the extent to which foveal tracking (through eye movements) or peripheral tracking (through off-target gaze fixation and peripheral vision) facilitates performance in the current task, is yet to be determined. The findings of Experiment 1 suggest an association between time spent looking at a screen region and tracking accuracy (Fig. 5). In Experiment 2, we aimed to extend this finding and assess the causal role of eye movements in the current trajectory tracking MOT task. We compared tracking performance of participants who freely moved their eyes during tracking (free-viewing), with those who kept their gaze fixed at the centre (fixed viewing).

## Experiment 2

This experiment aimed to investigate whether foveal or peripheral tracking of objects facilitates tracking performance in the current MOT task, as well as whether unequal attention allocation is possible with exclusive reliance on peripheral vision and covert attention. The critical role of peripheral vision has been identified in MOT tasks where equal attention allocation was required (Sears & Pylyshyn, 2000; Vater et al., 2016, 2017a, b). However, to our knowledge no study has explored peripheral tracking in a MOT task where unequal allocation of covert attention between screen regions is beneficial. In this study, priority was manipulated within subjects in the same way as in Experiment 1. The screen was divided vertically instead of horizontally to investigate whether the priority effects seen in Experiment 1 generalize to a different layout. Viewing condition was manipulated between subjects. Participants in the free-viewing condition were instructed that they were free to move their eyes around the screen during tracking, while participants in the fixed-viewing condition were instructed to keep their eyes fixated at the centre of the screen throughout the trial and track moving objects with their peripheral vision. The aims and hypotheses of Experiment 2 were preregistered on the Open Science Framework and can be found online (https://osf.io/bfje4/). Ethics approval was obtained from the School of Psychological Science Research Ethics Committee at the University of Bristol (113064).

## Method

### Participants

Sixty-Six individuals were recruited from the University of Bristol via the School of Psychological Science Experimental Hours Scheme, in return for course credit, and adverts on the School’s webpage, in return for the two highest achievers receiving a £50 Amazon voucher each. A top performer was identified from each viewing condition based on a performance score calculation (see below). Testing took place at the labs of the School of Psychological Science at the University of Bristol. For purposes of consistency with Experiment 1, a sample size of 66 participants was chosen (i.e., 33 participants in each of the two viewing conditions). With 66 participants, we had 80% power to detect an effect size of *dz* = 0.7, between the free viewing and fixed viewing conditions, at an alpha of 0.05. With 33 participants in each group, we should also have high power to detect an effect of priority within each group (given the size of this effect). Participants were required to have normal or corrected-to-normal vision and be less than 35 years old.

### Materials

The same apparatus as in Experiment 1 was used, apart from the following changes in the monitor and Eye Tracker. Stimuli were presented on a PC running Linux Mint 18 Sarah. A 24-in. ViewPixx 3D Lite monitor was used, with a resolution of 1,920 × 1,080 pixels, running at 120 Hz. The experiment was again run on a smaller screen window of 1,200 × 900 pixels. At a viewing distance of 70 cm, the display area subtends 46.6° × 24° and 1° corresponds to 45 pixels. An EyeLink 2000 (SR Research Ltd.) video-based tracker was used. The eyes were tracked at a sampling rate of 1000 Hz. The eye tracker was calibrated at the start of every block of trials (using the in-built 9-point calibration routine). Saccades and fixations were parsed offline using the velocity and acceleration criteria of 30°s^-1^ and 8000°s^-2^, respectively. Movement and appearance of the stimuli were identical to Experiment 1.

### Design

This study involved a mixed design. Priority was manipulated as a within-subjects factor with three levels (Low:30, Equal:50, and High:70) in the same way as in Experiment 1. Viewing-condition was manipulated as a between-subjects factor with two levels (free viewing versus fixed viewing). The same dependent variables as in Experiment 1 were used.

### Procedure

The MOT task used was identical to that of Experiments 1, yet in the current experiment the screen was divided vertically, instead of horizontally, in order to investigate whether the priority effect observed in Experiment 1 is replicated with a vertical screen division as well. The procedure regarding the number of practice and experimental trials, number of blocks and testing duration was identical to that of Experiment 1. In the fixed-viewing condition, participants were instructed to keep their eyes fixated at the centre of the screen throughout the tracking period. Compliance of participants with these instructions was encouraged by close monitoring of their eye movements by the experimenter, and regular reminders to keep fixating in the centre of the screen. At the end of every block, participants were provided with a score which reflected their performance on that particular block. This number represented the percentage of trials in which they specified a direction of movement which was within 20° of the correct direction of the item. This was done for purposes of participants’ compensation, to determine the two highest achievers (i.e., participants with the highest score) who would receive £50 each.

## Results

As in Experiment 1, we used LME analyses for the key perceptual and gaze measures. Priority (within-subjects continuous factor) and viewing condition (between-subjects categorical factor) were entered as fixed effects in the full model, along with their interaction. Both the null model and the full model allowed for a random intercept for subjects. For both perceptual performance and gaze measures, we compared the full model (including the predictor variables of priority and viewing condition, and their interaction) to the null model which included a random intercept for subjects only. The effects of priority, viewing condition, and their interaction are reported for every dependent variable from the full model, given that it was found to better predict the data compared with the null model. Data for both perceptual performance and gaze measures was analyzed aggregated across trials, to ensure normality of observations.

### Planned analyses

#### Perceptual performance

**Fig. 6:**
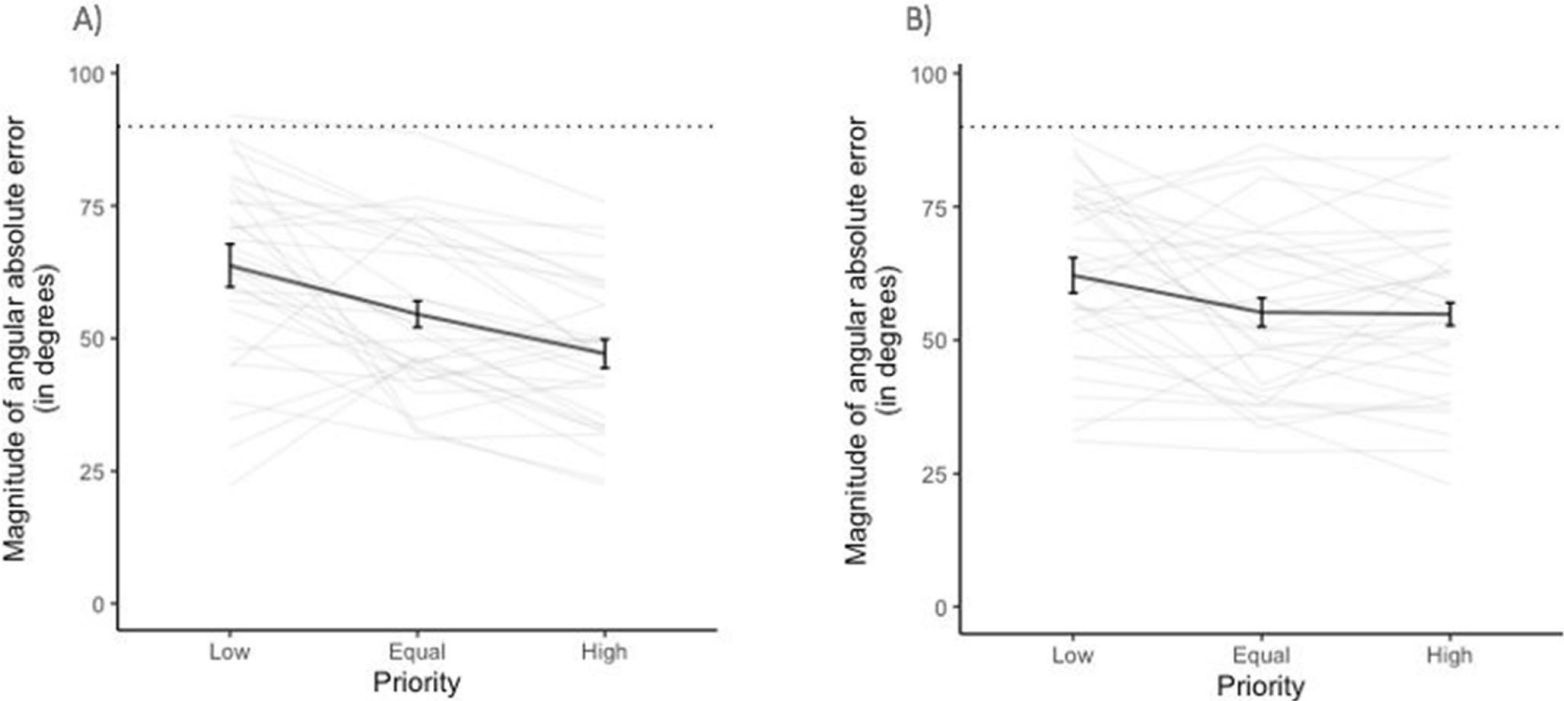
Magnitude of angular absolute error for each priority level in both free-viewing (**a**) and fixed-viewing (**b**) conditions. Black lines indicate average magnitude of angular error across all 33 participants in each viewing condition. Error bars represent 95% confidence intervals following Morey (2008). Grey lines indicate magnitude of angular error for each participant individually. Dashed horizontal lines indicate level of chance performance

Figure 6 indicates again better-than-chance tracking performance of participants in both free-viewing (Fig. 6a) and fixed-viewing conditions (Fig. 6b) across all three priority levels, suggesting that the task could be completed even when participants were not allowed to move their eyes. In both viewing conditions, there was better tracking accuracy in high versus low priority conditions. However, in the free-viewing condition, accuracy increased in a linear manner with priority. In contrast, in the fixed-viewing condition, there was similar tracking accuracy between equal and high priority conditions. The full mixed effects model (i.e., priority and condition as fixed effects along with their interaction) fit the data significantly better than the null model, χ^2^(3) = 46.7, *p* < .001. Specifically, priority had a significant effect on the magnitude of angular error, (*b* = −0.416, *SE* = 0.06, *t* = −6.82, *p* < .001). In both viewing conditions, as priority increased, the magnitude of angular error decreased. The viewing condition did not have a significant effect on the magnitude of angular error, (*b* = −9.422, *SE* = 5.38, *t* = -1.75, *p* = .082; Fig. 6). However, there was a significant interaction between priority and viewing condition (*b* = 0.234, *SE* = 0.086, *t* = 2.72, *p* = .007), suggesting that priority influenced magnitude of angular error differently across the two conditions, with participants demonstrating lower error in free-viewing (*M* = 47.12, *SD* = 49.61) compared with fixed-viewing (*M* = 54.90, *SD* = 53.66) conditions in the high priority screen regions (Fig. 6).

**Fig. 7:**
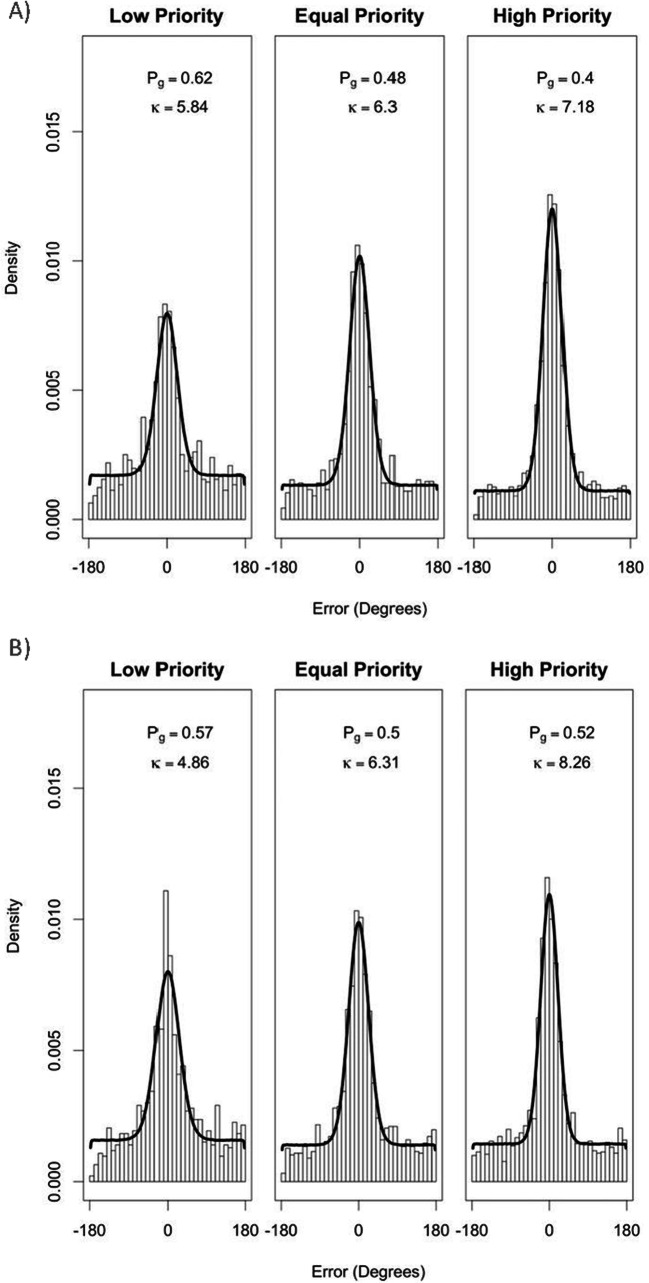
Mixture model fits for the three different priority conditions in free-viewing (**a**) and fixed-viewing (**b**) conditions of Experiment 2. The density plot demonstrates the actual data and the black density line illustrates the model fit. The best-fit parameters of the proportion of guessing (*P*_*G*_) and precision of tracking (*κ*) are also shown

Figure 7 shows the mixture model fits for the three different priority conditions in free-viewing (Fig. 7a) and fixed-viewing (Fig. 7b) conditions of Experiment 2. In line with initial predictions and the results of Experiment 1, model fits for the free-viewing condition are consistent with the analysis of error data for that condition (Fig. 6a): with increasing priority, tracking accuracy increased, the proportion of guessing decreased and precision increased.^4^ For the fixed-viewing condition, the model continues to capture the data well. However, no clear decreasing pattern of guessing is observed across the three priority conditions with similar guess rates in the equal priority condition and high priority conditions. This is expected, given the similar accuracy levels of participants observed in these two priority conditions (Fig. 6b). Precision increased as priority increased.

#### Gaze measures

**Fig. 8:**
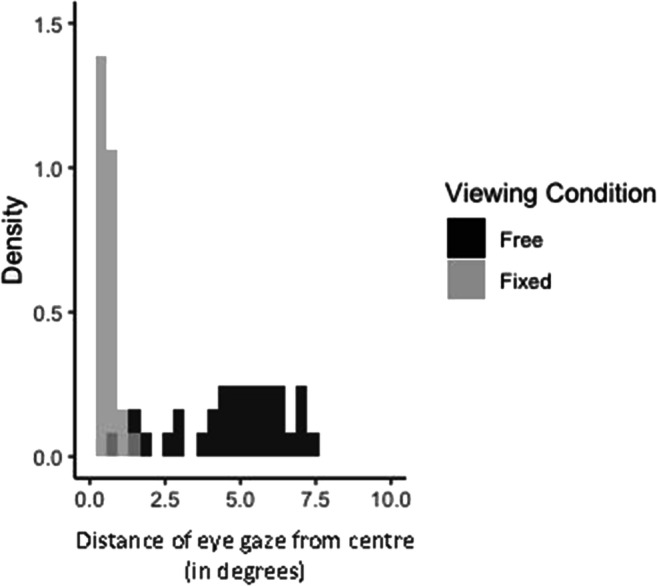
Average distance of each participant’s eye gaze from the centre in the two viewing conditions

Two measures were drawn from eye tracking data: proportion of time spent looking at the left screen region and the mean horizontal distance (in degrees) from the centre of the screen, at each of the three different priority levels (i.e., low, equal, or high). These measures were averaged across trials for each participant and analyzed with LME models in the same way as the angular error. First, however, we assessed the efficacy of the viewing condition manipulation to ensure that the participants’ eye movements matched the instructions they were given. Figure 8 indicates the average distance of eye gaze from the centre of the screen for each participant across the two viewing conditions. This was achieved by measuring the distance of each eye gaze sample from the centre of the screen, then calculating the average of those for each trial, and then across trials for each participant. We do not expect participants in the fixed-viewing condition to have an average distance of 0. Even if they complied with the instructions perfectly and never let their gaze move away from the centre by more than, say, a degree, we would expect small movements around fixation (micro-saccades and drift; Martinez-Conde et al., 2013; Rolfs, 2009). While there is some degree of overlap in the two distributions, it is clear that the majority of participants in the free-viewing condition moved their eyes around much more than participants in the fixed-viewing condition. Participants in the fixed-viewing condition also had much smaller variance in their distance travelled, as would be expected if they largely complied with the instruction to keep their gaze fixed.

**Fig. 9:**
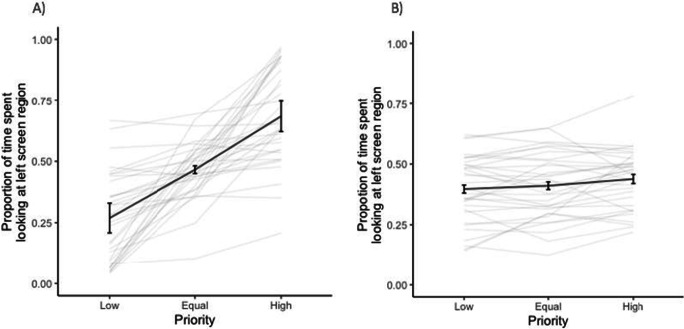
Proportion of time spent at the left screen region, for each priority level presented in both free-viewing (**a**) and fixed-viewing (**b**) conditions. Black bold lines indicate the average proportion of time spent looking at the left screen region across all 33 participants. Error bars represent 95% confidence intervals following Morey (2008). Grey lines indicate proportion of time spent looking at the left screen region for each participant individually

Figure 9 indicates the average proportion of time spent looking at the left screen region, as a function of priority of that area, across both viewing conditions. For the free-viewing condition, with increasing priority of the left region of the screen, participants spent more time in that region. However, in the fixed-viewing condition, roughly the same proportion of time was spent looking at the left screen region across all priority levels. To some extent, this result simply suggests that participants in the fixed-viewing condition complied with the instructions to fixate centrally. Nevertheless, we might have expected them to have subtle biases close to fixation, for example, through their microsaccades (Engbert & Kliegl, 2003). Such a bias would have shown up in this metric, because we simply tallied samples to the left and right of the vertical midline. Indeed, there *is *an overall bias toward fixating on the right side of the vertical midline, but this bias does not seem to be affected by priority.

The full LME model with priority and condition as fixed effects along with their interaction, fit the data significantly better than the null model, χ^2^(3) = 117.64, *p* < .001. Specifically, there was a significant effect of priority on proportion of time spent looking at the left screen region (*b* = 0.01, *SE* = 0.001, *t* = 13.32, *p* < .001). Also, there was a significant effect of viewing condition on proportion of time spent looking at the left screen region (*b* = 0.41, *SE* = 0.006, *t* = 6.68, *p* < .001). A greater proportion of time was spent looking at the left screen region in free-viewing (*M* = 0.48, *SD* = 0.24) compared with fixed-viewing condition (*M* = 0.42, *SD* = 0.14). Furthermore, there was a significant interaction between priority and viewing condition (*b* = −0.009, *SE* = 0.001, *t* = −8.47, *p* < .001).

The finding that participants in the free-viewing condition spent more time looking at a screen region that was more likely to be probed is further supported by the analysis of the mean horizontal distance of eye gaze from the centre (i.e., a + sign suggests leftward movement from the centre). Specifically, participants fixated further left from the centre when the left screen region was probed with a higher probability and further right from the centre when the left half was probed with a lower probability. The full LME model with priority and condition as fixed effects along with their interaction, fit these data significantly better than the null model, χ^2^(3) = 158.97, *p* < .001. In particular, there was a significant effect of priority of the left screen region on the mean horizontal distance from the centre, (*b* = 0.123, *SE* = 0.008, *t* = 15.53, *p* < .001). That is, with increasing priority at the left screen region, the mean horizontal distance moved towards the left side from the centre. These findings provide further evidence that participants’ gaze behaviour was influenced by priority, suggesting that participants were looking more at high versus low-priority regions, particularly when eye movements were permitted. Furthermore, there was a significant effect of viewing condition on mean horizontal distance from the centre (*b* = 6.305, *SE* = 0.59, *t* = 10.68, *p* < .001) presumably because participants in the free-viewing condition were on average further away from the centre. Finally, there was a significant interaction between priority and viewing condition (*b* = −0.1203, *SE* = 0.011, *t* = −10.72, *p* < .001) as participants’ distance from the centre in the free-viewing condition is likely to depend more on priority than in the fixed viewing condition.

### Exploratory analyses

**Fig. 10:**
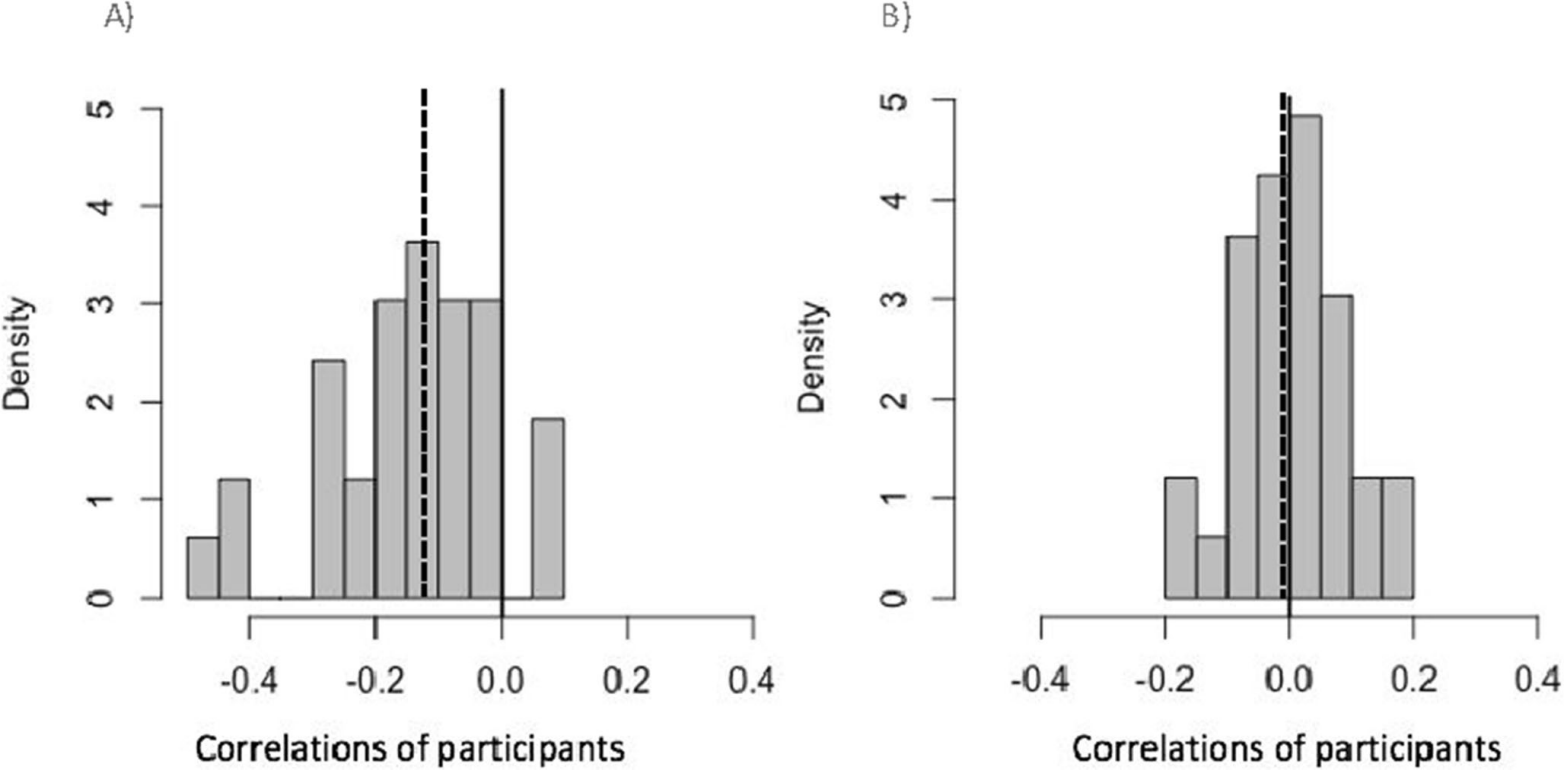
Histograms of correlations of individual participants between the proportion of time spent looking at the probed region and the tracking error (in degrees), in the free-viewing condition (**a**) and the fixed-viewing condition (**b**). The vertical bold lines indicate 0 correlation and the vertical dotted lines indicate the mean of all individual correlations

Similar to Experiment 1, we assessed the relation between the proportion of gaze time spent in the probed region with absolute tracking error, in both viewing conditions. We computed the correlation for each individual participant at a trial level, pooled over the three priority conditions. Figure 10a shows the distribution of participants’ correlations in the free-viewing condition where 91% of them were negative. The mean correlation of −0.15 in the free-viewing condition was significantly different from 0, *t*(32) = −6.03, *p* < .001. This indicates that in the free-viewing condition the more participants were looking in the probed screen region, the better their tracking performance (lower error). Figure 10b shows the distribution of correlations in the fixed-viewing condition where 48% of them were negative. It is clear that this distribution is much more symmetric around 0; indeed, the mean was −0.002 and was not significantly different from 0, *t*(32) = −0.13, *p *= .895.

**Fig. 11:**
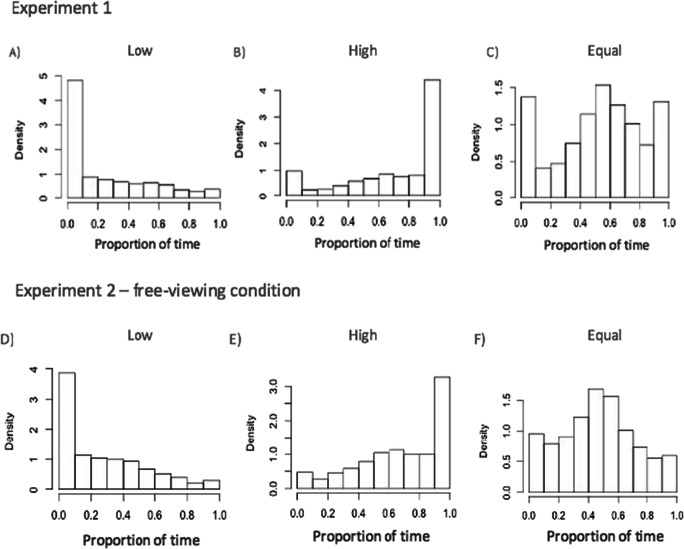
**a–c** Proportion of time spent looking at upper region of the screen on a trial level across all participants in Experiment 1, in low (**a**), high (**b**) and equal (**c**) priority conditions. **d–f** Proportion of time spent looking at left region of the screen on a trial level across all participants in free-viewing condition in Experiment 2, in low (**d**), high (**e**) and equal (**f**) priority conditions

One could argue that the effect of unequal attention allocation is not evident on every trial but is a result of the data being averaged across trials. This would mean that on some trials participants completely withdrew their attention from the low-priority region and tracked only objects in the high-priority region. According to this hypothesis, the probability matching happened across and not within trials (Eriksen & Yeh, 1985). To assess this possibility, we conducted an additional exploratory analysis of the proportion of time spent looking at each screen region *within* a trial for each priority condition. The between-trial probability matching account predicts that participants will spend almost all their time within a trial on one or the other region: for a given combination of region and priority, the distribution of the proportion of time spent in that region should have sharp peaks near 0 *and* 1, and very little density in between these extremes—in other words, a bimodal distribution. Within-trial probability matching predicts a unimodal distribution with a peak around the probability with which a region is probed. Figure 11 shows the proportion of time spent looking in the upper screen region in Experiment 1 (Fig. 11a–c) and at the left screen region in the free-viewing condition of Experiment 2 (Fig. 11d–f). These distributions are not consistent with the bimodal pattern predicted by between-trial probability matching, but they also do not completely fit the predicted pattern for within-trial probability matching. It is likely that there is a mixture of between and within-trial probability matching, where that mixture may result from between-participant differences in strategy or variations in strategy within participants over the course of the experiment. It is worth noting that participants’ performance was above chance levels in all three priority conditions which increases the likelihood of participants tracking both high and low tracking regions in most trials.

## Discussion

The perceptual report and eye-tracking results of Experiment 2 provide further evidence for unequal attention allocation across screen regions, in both fixed- and free-viewing conditions. On the one hand, in the free-viewing condition, behavioural findings illustrate that participants have improved tracking accuracy as priority increased (Fig. 6). Eye-tracking findings support this, showing an increased proportion of time looking at a high priority screen region (Fig. 9) and greater distance from the vertical midline towards the high priority side as the priority presented in that region increased. These findings indicate that participants were allocating their attention unevenly across screen regions, based on the probability of a probe occurring in that region, supporting the results of Experiment 1. On the other hand, in the fixed-viewing condition, as participants fixated at the centre, roughly an equal proportion of their eye gaze was spent on each screen region. Interestingly, even with this eye movement pattern, which is more concentrated around the centre, participants’ tracking performance improved as priority increased (Fig. 6). This indicates that even without eye movements and by relying exclusively on peripheral vision, unequal attention allocation is still possible yet, only to a certain extent. Similar tracking performance was observed in the equal and high priority conditions in the fixed-viewing condition, suggesting that with covert attention, unequal attention allocation is possible in a less fine-grained manner compared with when foveal vision is employed (Fig. 6). Overall, the findings of Experiment 2 suggest that eye movements play a causal, albeit modest, role in improving perceptual performance in the high-priority region.

## General discussion

In the present study, we aimed to investigate unequal attention allocation between two distinct regions and the extent to which unequal allocation relies on eye movements (i.e., overt attention). Taken together, the findings of Experiment 1 and 2 provide evidence of unequal attention allocation between two distinct visual regions in the current MOT task. Tracking accuracy improved with increasing probability of a region being probed and participants fixated more in the high-priority region. Participants in both experiments were even found to allocate their attention between the two screen regions proportionally based on the priority of each region. In particular, roughly 70% of their time was spent on the high-priority regions, 50% of their time was spent on the equal priority regions, and about 30% of their time was spent on the low-priority region. This finding is reminiscent of probability matching (Eriksen & Yeh, 1985) and supports the idea that participants devoted the majority of their attention to the high-priority region but did not completely neglect the low-priority regions. Evidence of goal-directed unequal allocation of attention was also obtained under the fixed-viewing condition of Experiment 2, where moving objects were tracked solely with peripheral vision. This finding indicates that unequal attention allocation is possible without eye movements, although priority had a greater and more fine-grained effect on tracking performance in the free-viewing condition. Therefore, we conclude that unequal attention allocation is possible even when relying on peripheral vision. Nonetheless, eye movements, when permitted, do improve tracking accuracy, as participants are able to fixate in the high-priority region and get a more precise foveal view of moving targets (Landry et al., 2001).

Compared with Crowe et al. (2019), we observed higher guessing rates in both Experiments 1 and 2 and worse tracking performance, which can probably be attributed to the different demands of the two tasks, resulting in different levels of difficulty. In the current modified MOT task, participants did not know from the beginning of the trial which of the four objects in each screen region would be the target—that is, which object they would be questioned about. Therefore, participants had to track all eight objects across the two regions, which may have well increased the difficulty of our task compared with Crowe et al. (2019) in which only two objects needed to be tracked among six distractors. Tracking eight objects is likely to exceed the capacity limits for most observers (typically estimated around four objects; Intriligator & Cavanagh, 2001; Scholl et al., 2001). To cope with the high tracking load it is possible that participants simply drop tracking of some objects, probably from the low-priority region and focus primarily on tracking of objects in the high-priority region. This explanation is supported by the increased guessing rates of participants in the low-priority regions. Nevertheless, both perceptual performance as well as gaze measures warrant against complete dropping of *all* low-priority targets as tracking accuracy is well above chance tracking performance and participants are found to spend a significant amount of time looking at low-priority regions. Participants might have dropped tracking of *some* targets from the low-priority regions yet, given that the priority is associated with the whole screen region and not individual targets, we believe that dropping tracking of *some* targets in line with priority, is a form of unequal attention allocation.

The findings of the current experiments provide further support to existing literature demonstrating that top-down instructions can guide goal-directed attention allocation (Brockhoff & Huff, 2016) and can be used to manipulate attention allocation of observers in different MOT tasks (Crowe et al., 2019; Cohen et al., 2011; Fitousi, 2016; Miller & Bonnel, 1994; Yantis, 1992). It appears that attention can be allocated adaptively across objects in different regions of the visual field depending on task demands. Additionally, the findings of Experiment 2 are in line with the literature on the usefulness of peripheral vision (Vater et al., 2016, 2017a, b) in attentional tracking, and extend this view by showing that covert attention can be allocated *unequally* between different tracking regions, although not in a very fine-grained manner. Nevertheless, the tracking advantage of foveal vision observed in the high priority condition of Experiment 2 suggests that overt attention is more effectively allocated unequally than covert attention. It is worth noting that past findings on the use of overt and covert attention during tracking were obtained from traditional MOT tasks in which attention was, presumably, allocated equally to targets. Having established the plausibility of unequal attention allocation using both overt and covert attention in the current series of experiments, future work should investigate specific tracking strategies (Fehd & Seiffert, 2008, 2010; Zelinsky & Neider, 2008) used during *unequal* attention allocation to investigate *how* exactly observers distribute their attention unevenly across different targets or regions of the visual field. For example, rather than centroid tracking (fixating in at the centre of the mass of targets) participants might be biased off centroid towards higher priority targets.

From an applied point of view, the current modified MOT task allows for an investigation of *unequal* attention allocation, better reflecting situations outside of the laboratory where observers are required to allocate their attention unevenly between different targets or regions of the visual field (e.g., a goal keeper having to track movement of more than one player or a security guard having to track movement of multiple people in different CCTV monitoring screens). Specifically, in both sports (Abernethy et al., 2001; Ward et al., 2002) and driving (Deng et al., 2019; Kotseruba et al., 2016; Wong & Huang, 2013) settings, information in different locations can vary in importance so attention needs to be allocated unequally in order to make a good judgment. Insights into how effectively attention can be allocated across these regions is, therefore, potentially valuable in informing practice in professional tasks that require attention to be allocated across multiple regions in an unequal manner.

Findings regarding the use and efficacy of peripheral vision compared with foveal vision can be applied in the context of driving and sports as well, given that peripheral vision is extremely important in both settings. For instance, sports players are often found to focus their eye gaze on an anchor point located between different visual regions of interest and process information from each area using peripheral vision (Milazzo et al., 2016; Piras & Vickers, 2011; Vansteenkiste et al., 2014), while drivers are found to use their peripheral vision for hazard perception and maintaining lane position (Costa et al., 2018; Summala et al., 1996). Therefore, based on the plausibility of unequal attention allocation (see also Chen et al., 2013; Crowe et al., 2019; Liu et al., 2005) and the functionality of peripheral vision in detecting motion changes during MOT (Fehd & Seiffert, 2008, 2010; Vater et al., 2016, 2017a, b; Zelinsky & Neider, 2008), gaze-strategy training programmes in sports and driving contexts can be designed more carefully by taking into consideration the different capabilities of the human visual system. Given that driving accidents have been associated with misallocation of visual attention (Dingus et al., 2006; Klauer et al., 2006; Lee, 2008) it is highly important to invest in improving visual attention skills of drivers to increase road safety.

An important consideration of the modified MOT task used in the current experiments is the identical nature of all moving objects (i.e., 8 identical discs), which is unlike real-world settings were the individual targets we are tracking all have unique identities. Future research should therefore examine the role of foveal and peripheral vision in unequal attention allocation in a MIT task, where individual objects will have unique identities (Oksama & Hyönä, 2008). For example, the current modified trajectory-tracking MOT task can be altered into a trajectory-tracking MIT task where images of real objects (Iordanescu et al., 2011; Oksama & Hyönä, 2016) could be used instead of black discs. Such an investigation would shed light on how different target properties, in terms of similarity structure and saliency, can influence tracking strategies used by observers when dividing their overt and covert attention unequally across targets with unique identities.

The current study provides evidence of unequal attention allocation between two distinct tracking regions in a modified trajectory-tracking MOT task in a top-down fashion. These findings were obtained under both free-viewing and fixed-viewing conditions, indicating that unequal attention allocation is possible without eye movements. However, when permitted, eye movements improve accuracy, as participants are able to focus their gaze on the region with the highest priority. Having established the plausibility of unequal attention allocation using both overt and covert attention, this study offers an insight into the functional role of eye movements during attentional tracking. The incorporation of eye-tracking methods when investigating unequal attention allocation in future experiments should further clarify the current findings and shed light on *how* exactly attention is allocated across different targets or regions of the visual field.

## Data Availability

The datasets generated and analyzed during the current study are available on the Open Science Framework (OSF) page of each experiment (Experiment 1 of manuscript: https://osf.io/wkcj5/files/; Experiment 2 of manuscript: https://osf.io/bfje4/files/). The aims and hypotheses of Experiment 1 were preregistered on the OSF and can be found at: https://osf.io/wkcj5/. The aims and hypotheses of Experiment 2 were preregistered on the Open Science Framework and can be found at: https://osf.io/bfje4/.

## Footnotes

1 Note that there is a discrepancy between the paired comparisons assumed in the power calculation and the LME adopted for the actual analysis. Therefore, we performed a power calculation in R using the SIMR package suitable for an LME design (Green & Macleod, 2016). With an effect size of priority of −0.44 (derived from a pilot experiment), a sample of 33 participants gives us at least 99% power of detecting a similar effect at an alpha of.05.

2 The number of trials from each trial type was incorrectly described in the preregistration protocol.

3 When model analysis was done on individual participants, the effects of priority on the parameters of proportion of guessing and tracking precision were more fragile, presumably because more data would be needed to estimate the model parameters reliably for each participant individually.

4 Similar to Experiment 1, when model analysis was done on individual participants, the effects of priority on the parameters of proportion of guessing and tracking precision, were more fragile, probably because more data would be needed to model parameter estimates for each participant individually.

## Code availability

Code for the main analysis of the three dependent variables (i.e., angular error; proportion of time spent on top/left screen regions; mean vertical/horizontal distance from centre) and analysis of mixture mode fits, for Experiments 1 and 2 can be found on the OSF page of each project (https://osf.io/wkcj5/files/;https://osf.io/bfje4/files/, respectively). MATLAB code for running these experiments and the rest of the analysis code is available upon request.
